# Supplementary material for: JAK1/2 Inhibition Delays Cachexia and Improves Survival through Increased Food Intake
Source: bioRxiv. 2025 Oct 22:2025.10.21.683287. Preprint. [Version 1] doi: 10.1101/2025.10.21.683287 (PMC12633540; doi:10.1101/2025.10.21.683287)
Supplement: 1 [file NIHPP2025.10.21.683287V1-supplement-1.pdf]

bioRxiv preprint doi: https://doi.org/10.1101/2025.10.21.683287; this version posted October 22, 2025. The copyright holder for this preprint (which was not certified by peer review) is the author/funder, who has granted bioRxiv a license to display the preprint in perpetuity. It is made available under aCC-BY 4.0 International license.

Supp. Fig. 1

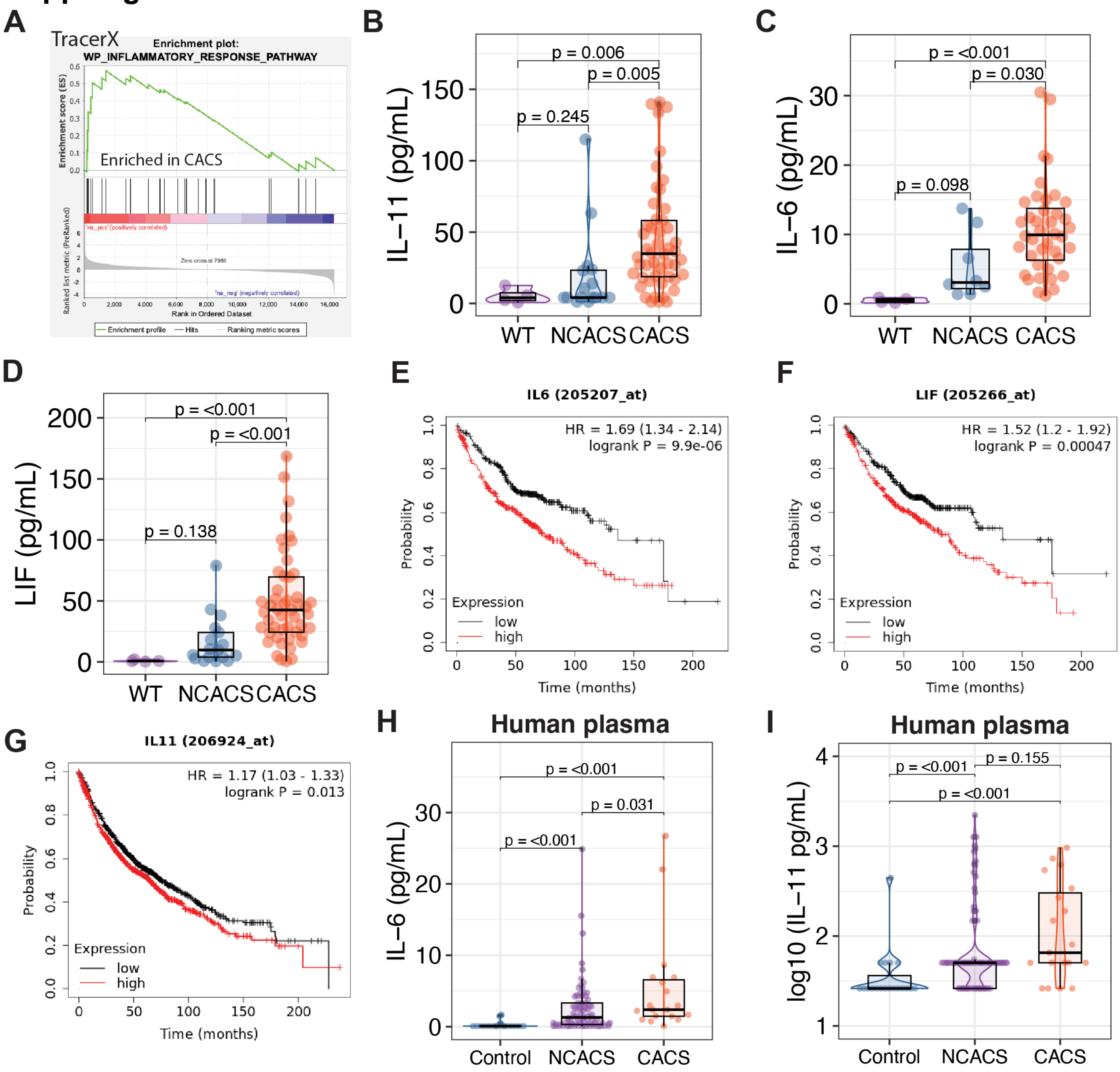

Supp.Fig.1. IL-6 family members are associated with an unfavorable prognosis in human lung cancer.

A. GSEA comparing CACS to NCACS in tumor transcriptomics from the TracerX cohort, showing increased inflammatory response in the tumors of patients with cachexia. B-D. Circulating levels of IL-11 (B), IL-6(C) and LIF(D) in non-tumor bearing mice (WT), NCACS and CACS mice. **B-D.** Kaplan-Meier plots showing survival probability of patients with lung adenocarcinoma stratified by high or low tumor expression of IL-6, LIF and IL-11. **E and F.** Protein concentration measured by Luminex of IL-6(E) and IL-11(F) in the plasma of lung cancer patients with and without cachexia and control plasma samples. Comparisons in E and F were done with the Kruskal-Wallis test, followed by Dunn's test. Individual data points are independent biological replicates unless otherwise stated.

bioRxiv preprint doi: https://doi.org/10.1101/2025.10.21.683287; this version posted October 22, 2025. The copyright holder for this preprint (which was not certified by peer review) is the author/funder, who has granted bioRxiv a license to display the preprint in perpetuity. It is made available under aCC-BY 4.0 International license.

Supp .Fig 2

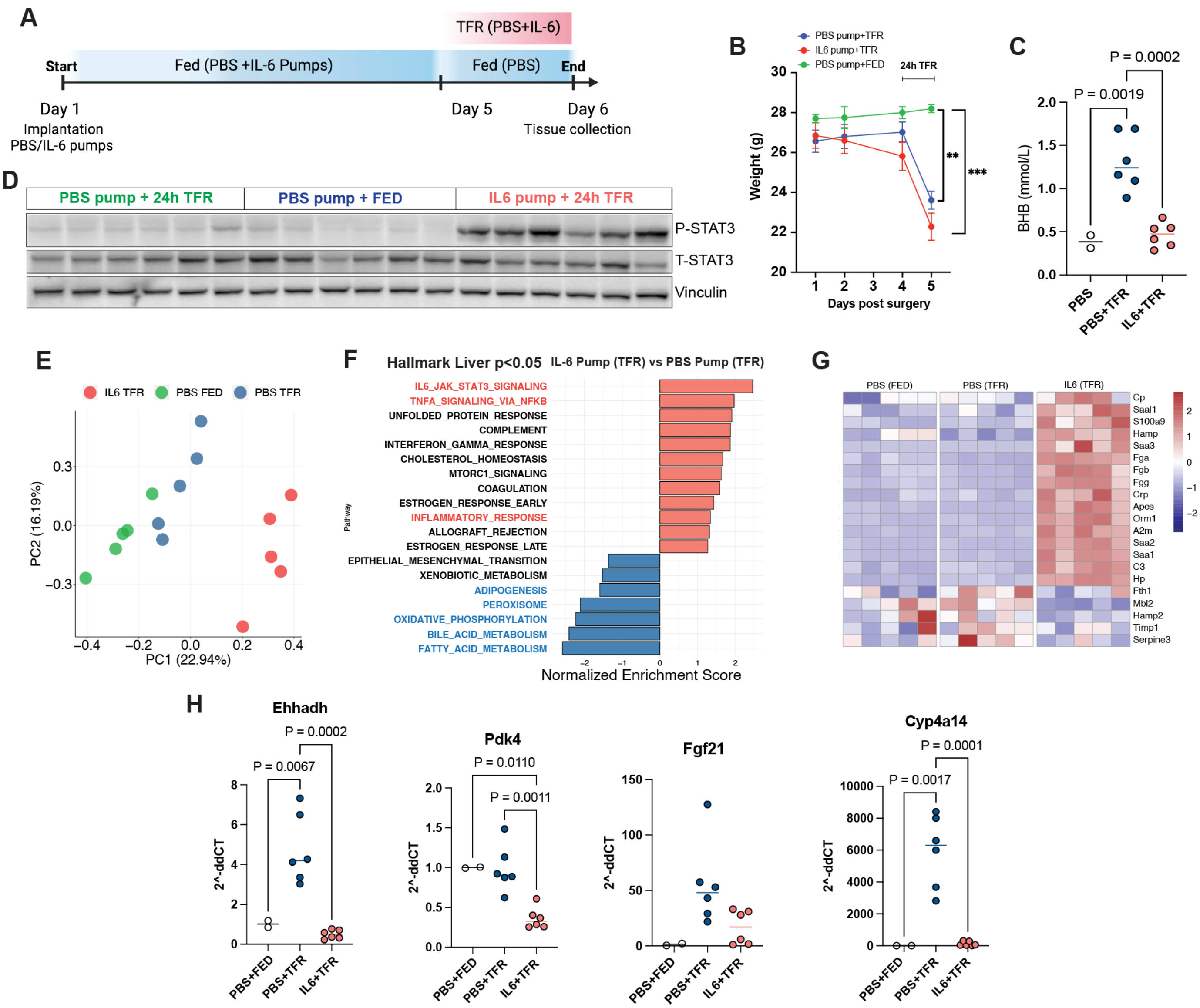

Supp.Fig.2. IL-6 reproduces the liver phenotype of the KL CACS mice.

A. Experimental design for non-tumor bearing mice implanted with IL-6 or PBS secreting pumps (“Fed” is unrestricted access to food, “TFR” is total food restriction for 18 hs). **B.** Whole-body weight of mice implanted with IL-6 secreting pumps and total food restriction for 24hs (TFR, red), mice with PBS pumps and 24hs TFR (blue), and PBS pumps with ad-libitum food (green). **C.** Beta-hydroxybutyrate (BHB) measured in the serum of the mice in A/B. **D.** WB for p-STAT3 (Tyr705), Total STAT3 and Vinculin in the liver of mice implanted with PBS or IL-6 pumps **E.** Unbiased principal component analysis (PCA) of liver transcriptomics from WT (non-tumor bearing) mice implanted with IL-6 secreting pumps and total food restriction for 24hs (TFR, red), mice with PBS pumps and 24hs TFR (blue), and with PBS pumps but with ad-libitum food (green). **F.** GSEA of liver transcriptomics comparing Hallmark pathways between mice implanted with IL-6 or PBS-secreting pumps and TFR for 24hs. **G.** Heatmap of APR-related genes in the liver transcriptomics of mice implanted with PBS or IL-6 pumps. **H.** qPCR for PPAR-α target genes in livers of mice implanted with PBS or IL-6 pumps. Comparisons in C and H were performed with one-way ANOVA followed by Tukey's multiple comparisons test. Comparisons in J were done using a two-tailed Student's t-test. Comparisons in B were done with two-way ANOVA followed by Tukey's multiple comparisons test (**=0.0008, ***=<0.0001). Individual data points are independent biological replicates unless otherwise stated. Model in (A) was made with Biorender.com.

bioRxiv preprint doi: https://doi.org/10.1101/2025.10.21.683287; this version posted October 22, 2025. The copyright holder for this preprint (which was not certified by peer review) is the author/funder, who has granted bioRxiv a license to display the preprint in perpetuity. It is made available under aCC-BY 4.0 International license.

Supp .Fig 3

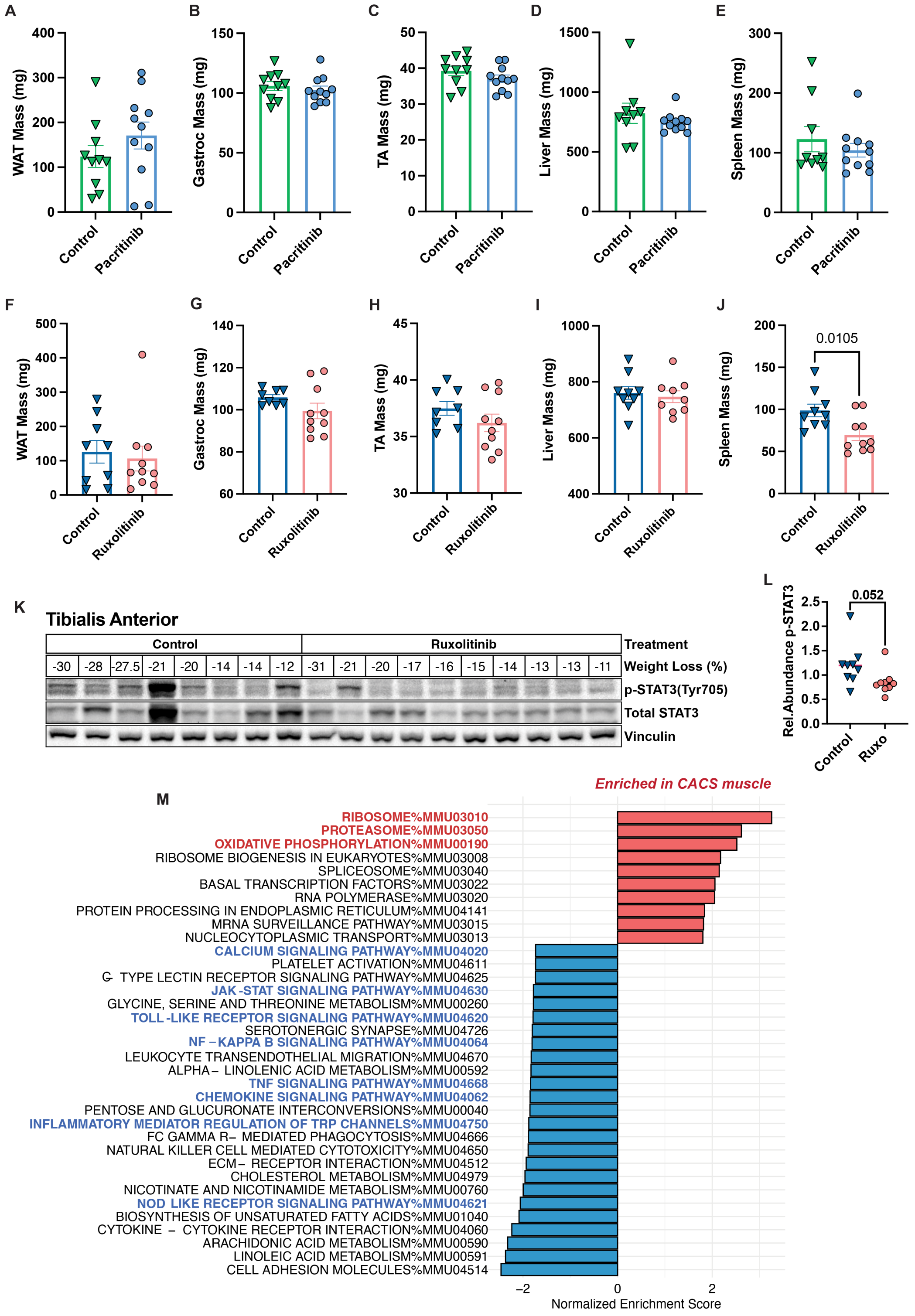

Supp.Fig.3. Effects of JAK/STAT3 inhibition in peripheral tissues.

A-B. Averaged tissue weights from KL mice at endpoint treated or not with pacritinib. **C-D.** Averaged tissue weights from KL mice at endpoint treated or not with ruxolitinib. **E.** WB for p-STAT3 (Tyr705), Total STAT3 and Vinculin in the tibialis anterior of KL male mice treated or not with ruxolitinib. **F**. Quantification by densitometry of the p-STAT3 WB shown in (K). **G.** GSEA using the Kyoto Encyclopedia of Genes and Genomes (KEGG) database to compare muscle from cachexic mice treated or not with ruxolitinib (q-val <0.001). Comparisons between control and pacritinib or ruxolitinib-treated mice in A-J and L were done using a two-tailed Student's t-test. Individual data points are independent biological replicates unless otherwise stated.

bioRxiv preprint doi: https://doi.org/10.1101/2025.10.21.683287; this version posted October 22, 2025. The copyright holder for this preprint (which was not certified by peer review) is the author/funder, who has granted bioRxiv a license to display the preprint in perpetuity. It is made available under aCC-BY 4.0 International license.

Supp. Fig. 4

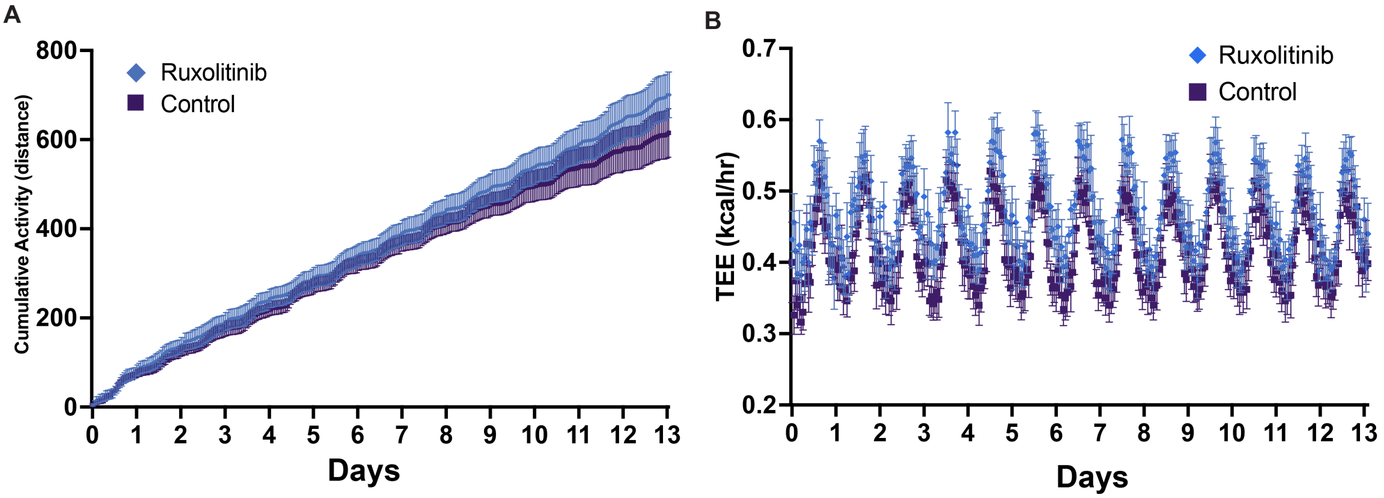

Supp.Fig.4. Ruxolitinib does not affect activity or energy expenditure in non-tumor bearing mice. A. Cumulative activity of non-tumor bearing mice treated or not with ruxolitinib. B. Total energy expenditure of non-tumor bearing mice treated or not with ruxolitinib.
